# Personals

**Published:** 1913-05

**Authors:** 


### PERSONALS.

Dr. L. E. Curtice of Buffalo will remove May 1 to 346 Massachusetts street.

Dr. Joseph Roby of Rochester moved April 1 to 234 Culver Road, corner of Park avenue.

Dr. Edward L. Hanes of Rochester has moved to 748 East Main street. His practice is limited to Nervous and Mental diseases.

Dr. A. W. Booth of Elmira has returned from a month's trip to Cuba and Panama.

Dr. Charles Haase of Elmira attended the meeting of the Metropolitan Medical Examiners in Utica in March.

Dr. E. R. Linklater of Buffalo will move May 1 to 233 W. Utica street.

Dr. George E. Fell of Buffalo has moved to 802 D. S. Morgan Building.

Dr. Herman E. Hayd of Buffalo returned early in April from a Panama trip.

Dr. William Stanton, Webster, N. Y., U. B., '89, announces his removal, April first, to his new residence at Main street and Dunning avenue, where he has a five-room office, including operating room and laboratory, equipped with modern conveniences for medical and surgical work.

Dr. Joseph Brennan, a former Buffalo resident, has returned from New York and has located at 399 Delaware avenue.

Dr. W. E. Lundblad, Director of the Steuben County Bacteriologic Laboratory, has resigned.

Dr. H. M. Cowper of Buffalo has moved to the Vedder house, 543 Franklin street.

Dr. Thomas Walsh of Buffalo has returned from an extended trip to Europe.

Dr. Flavel B. Tiffany of Kansas City has returned from a trip around the world. He made a special study of the Indian operation for cataract with the originator, Col. Smith of Bombay.

Dr. Lee Adrian Whitney of Rochester announces the removal of his office to 255 Alexander street.

---

Dr. J. C. Clemesha of Buffalo has gone to Europe.

---

Dr. S. Y. Howell of Buffalo, after returning from Panama, will make a tour around the world.

---

Dr. Mabel Flood of Elmira visited Dr. Regina Flood Keyes of Buffalo late in March.

---

Dr. John H. Grant, formerly of Buffalo, has recently been admitted to membership in the Medical Society of the County of New York.

---

Dr. Zoena M. Sutton of Tonawanda is studying at the College of Missions in Indianapolis, in preparation for work in India.

---

Dr. W. H. Annowski of Buffalo returned from Florida in March and is now studying in New York City.

---

Dr. Joseph S. Lewis of Buffalo has removed to 11 Irving Place.

---

Dr. James F. Rice of Buffalo has moved to 441 Elmwood avenue.

---

The Pennsylvania State College Alumni Association of Western New York held its banquet in Buffalo, March 28, Dr. H. H. Glosser presiding. The President of the College, Dr. Edwin E. Sparks, was present.

---

Dr. Roland Meisenbach of Buffalo has recently returned from a western tour, including Kansas City and St. Louis. In Kansas City he held a clinic and in the evening spoke to the Jackson Co. Medical Association on the Sacro-Iliac joint. In St. Louis he spoke before the St. Louis Medical Society on "Abbott's New and Rapid Method of Correcting the Fixed Types of Lateral Curvature." During his stay in St. Louis a number of banquets were given in his honor.

---

Dr. Russel H. Wilcox of Delaware street, Tonawanda, who was recently appointed as second lieutenant of Company K, 74th Regiment, was sworn in April 14.

Dr. Louis Hartman, Surgeon of the N. Y. C. & H. R. R. R. at Syracuse, has recently moved to Detroit.

Dr. Leighton R. Cornman of Rochester has moved to 266 Alexander street.

